# Protective Effect of *Hibiscus Sabdariffa* on Doxorubicin-induced Cytotoxicity in H9c2 Cardiomyoblast Cells

**Published:** 2017

**Authors:** Azar Hosseini, Elham Bakhtiari, Seyed Hadi Mousavi

**Affiliations:** a *Pharmacological Research Center of Medicinal Plants, School of Medicine, Mashhad* *University of Medical Sciences, Mashhad, Iran. *; b *Eye Research Center, Mashhad University of Medical Sciences, Mashhad, Iran. *; c *Clinical Research Development Unit, School of Medicine, Mashhad University of Medical Sciences, Mashhad, Iran. *; d *Medical Toxicology Research Center, School of Medicine, Mashhad* *University of Medical Sciences, Mashhad, Iran.*

**Keywords:** *Hibiscus sabdariffa*, H9c2 cells, Doxorubicin, Apoptosis

## Abstract

Doxorubicin (DOX) is an effective anticancer drug. But its clinical application is limited, because DOX induces apoptosis in cardiomyocytes and it leads to permanent degenerative cardiomyopathy and heart failure. Recent trainings showed that *Hibiscus sabdariffa *exhibit pharmacological actions such as potent antioxidant. So, in this study we explored the protective effect of *H. sabdariffa* extract on doxorubicin-induced cytotoxicity in H9c2 cells. Cell viability was quantified by MTT assay. Apoptotic cells were determined using PI staining of DNA fragmentation by flowcytometry (sub-G1 peak). Cells were cultured with 5 μM DOX for 24 h to create the cell damage. H9c2 cells were pretreated with different concentrations (7.81-500 μg/mL) of *H. sabdariffa *extract (HSE) for 2 h before DOX treatment in all trials. Pretreatment with HSE increased cell viability at concentration of 31.25-500 μg/mL. Compared to control cells, apoptosis was induced in DOX treated cells after 24 h, (𝑃< 0.001). Pretreatment with HSE significantly decreased cell apoptosis after 24 hr at concentration of 31.25-250 μg/mL.

Our results show that *H. sabdariffa* could exert the cardioprotective effects on DOX-induced toxicity partly by antiapoptotic activity.

## Introduction

Doxorubicin (DOX) is an effective cytotoxic antibiotic that is used in treatment of several cancers such as leukemia, lymphoma, breast, lung, and other solid tumors (1). But its clinical use is limited because it has serious irreversible cardiotoxicity and even can lead to heart failure in a dose-dependent manner (2). The exact mechanism of DOX-induced cardiotoxicity is not understood, but many reports indicate that generation of free radicals and reactive oxygen species (ROS) has a major role in this toxicity (2-3). Free radicals are being generated in the body as a result of common metabolic processes but DOX led to produce excess amount of ROS and impairment of cardiac cell function (2). 

Researches show that natural products with antioxidant activity can improve or prevent oxidative damage of DOX. For example, survivin (4), sesamol (5), and Herba leonurine (6) could protect myocytes against cardiotixicity of DOX.* Hibiscus sabdariffa* (also famous as sour tea) from the Malvaceae family is cultured and grown naturally in tropical regions including south of Iran. This plant has been used in different countries as a cooking and curative substance. The obese fruiting calyces of this plant have been used for preparing candies and beverages. In traditional medicine, the calyx extract of this plant is used for treatment of several diseases, including liver diseases (7), high blood pressure (7), cardiovascular disease (8) and atherosclerosis (8).

**Figure 1:**
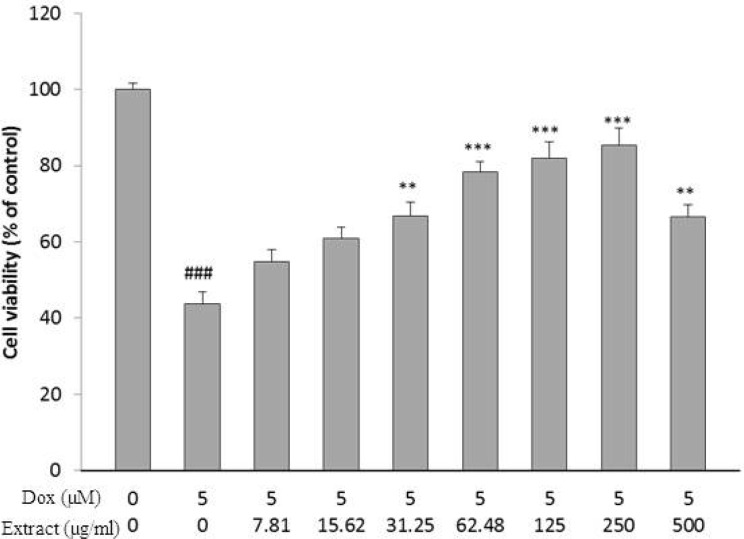
Effect of HSE on H9c2 cells viability exposed to DOX for 24 h. The percentage cell viability (quantitated by MTT assay) was normalized against the control.^ ###^𝑃 < 0.001 versus control, *** *P *< 0.001, ** *P *< 0.01 DOX

**Figure 2 (a,b).**
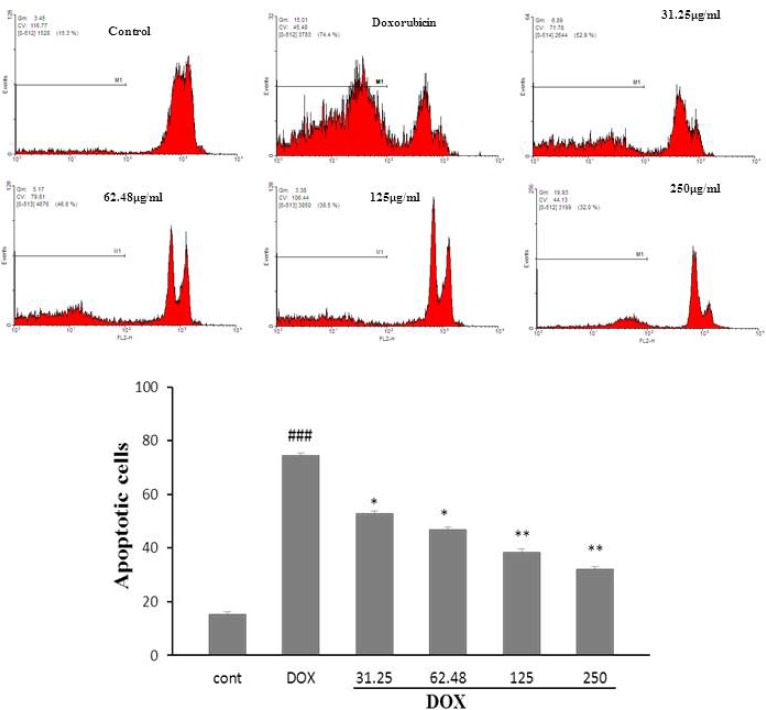
The effects of the HSE on apoptosis in H9c2 cells using PI staining and flow cytometry. ^###^𝑃 < 0.001 versus control, ^**^𝑃 < 0.01and ^*^𝑃 < 0.5 versus DOX

The chemicals existing in the flowers of *H.sabdariffa* includes anthocyanins (delphinidin-3-glucoxyloside,delphinidin- entoside-glucoside, delphinidin-3-ambubioside, cyanidin-monoglucoside,cyanidin-3,5-diglucoside, cyanidin-3-sambubioside, cyanidin-3-glucosylrutinoside,cyanidin-3-glucoside), flavonol glycoside,gossypitrin, sabdaretin, myricetin, hibiscetrin, quercetin, luteolin, a luteolin glycoside, chlorogenicacid, flavonoids (gossypetin, hibiscetin, and their respective glycosides), and protocatechuic acid (7). Almost all of these chemical ingredients have potent antioxidant properties. Several studies reported that HSE has potent antioxidant properties and has great capacity for scavenging free radicals (9-10). As it has been discovered that *H. sabdariffa *has many valuable consequences correlating with its antioxidant effect, we decided to train its protective effect on the doxorubicin-induced cytotoxicity in H9c2 cells.

## Material and Methods

*Cell Line and Substances*

The rat heart-derived myoblast cell line; H9c2 cardiac myocytes was purchased from Pasteur Institute (Tehran, Iran). Glucose-high Dulbecco’s modified Eagle’s medium (DMEM), fetal bovine serum (FBS), and penicillin streptomycin were purchased from Gibco (Grand Island, NY). Dimethyl sulfoxide (DMSO) was purchased from Merck. Propidium iodide (PI), sodium citrate ,Triton X-100, Dulbecco’s Phosphate-buffered saline (PBS), doxurubicin, and 4, 5-Dimethylthiazol-2-yl, 2, 5-diphenyl tetrazolium (MTT) were purchased from Sigma (St Louis, MO, USA).

*Cell Culture*

H9c2 cells were cultured in high glucose DMEM (4.5 g/L) supplemented with 10% FBS and 100 units/mL of penicillin/streptomycin. All cells were maintained in a humidified atmosphere (90%) containing 5% CO2 at 37 ^0^C.

*Preparation of Hibiscus sabdariffa extract (HSE)*

The calyces of *H. sabdariffa* were collected from the traditional market of Mashhad, Iran. The calyces were dried, powdered and subjected to extraction with 70% ethanol in a Soxhlet apparatus for 48 h. The HSE was then dried on a water bath and the yield (24% w/w) dissolved in DMSO.

*Cell Proliferation Assay (MTT)*

H9c2 cells (5000/well) were seeded out in 96-well culture plates. Different concentrations of HSE were performed 2 h before DOX treatment in all experiments. Cells were cultured with 5 μM DOX for 24 h to make the cell injury model. For MTT test, after removing the medium, MTT solution (5 mg/mL in PBS) was added for 1.5 h resulting formazan, which was solubilized with DMSO (100 *μ*L) (11). The absorbance at 570 and 620 nm (background) was measured using a StatFAX303 plate reader. 

*Cell apoptosis assay*

Apoptotic cells were known by using PI staining of small DNA fragments followed by flow cytometry. It has been reported that a sub-G1 peak that is reflective of DNA fragmentation can be observed following the incubation of cells in a hypotonic phosphate-citrate buffer containing a quantitative DNA-binding dye, such as PI. Apoptotic cells that have losing DNA will take up less stain and appear on the left side of the G1 peak in the histogram. 

At first, H9c2 cells were seeded in 24-well culture plate and after 24 h the cells were pretreated with HSE (7.81-500 µg/mL) for 2 h and then incubated for 24 h with DOX for cell injury. Floating and adherent cells were then harvested and incubated at 4 °C overnight in the dark with 750 µL of a hypotonic buffer (50 µg/mL PI in 0.1% sodium citrate with 0.1% Triton X-100). Next, flow cytometry was carried out using a FACS that can flow cytometer (Becton Dickinson). A total of 104 events were acquired with FACS.

*Statistical Analysis*

One-way analysis of variance (ANOVA) was utilized for data analysis. All results were expressed as mean *±* SEM. *P < *0*.*05 was considered statistically significant.

## Results

*HSE Dose-Dependently Inhibits DOX Induced Cell Death*

To clarify the probable toxic effects of HSE, H9c2 cells were incubated with diverse concentration of HSE (7.81-500 µg/mL), and the cell viability was determined 24 h after treatment. No significant toxic effect on cell viability was seen in the following treatment with HSE for 24 h.

DOX caused a major decrease in cell viability after 24 h, as compared with control group (𝑃< 0.001, Figure 1). As shown in Figure 1, pretreatment with HSE as concentration and time dependently increase cell viability following DOX insult for 24 h (𝑃< 0.01 at 31.25 µg/mL and 500 µg/mL) and (𝑃 < 0.001 at 62.48-250 µg/mL)

*HSE Significantly Decreases DOX-Induced cell apoptosis*

The results revealed that exposure of H9c2 cells to DOX, significantly augment cell apoptosis, compared with control group (𝑃 < 0.001, Figure 2a,b). A significant decrease in DOX-induced apoptosis was seen following pretreatment with HSE (31.25 and 62.48 µg/mL, 𝑃< 0.5; 125 and 250 µg/mL, 𝑃 < 0.01) 

## Discussion

Doxorubicin is one of the most commonly used chemotherapy drugs, but its toxicity on myocytes has been a major distress in clinical use for years. Doxorubicin-induced cardiotoxicity causes cardiac myocyte loss, cardiomyopathy, and heart failure (12). Although several researches on doxorubicin-induced cardiotoxicity have been performed over the years, the principal mechanisms responsible for doxorubicin-induced cardiotoxicity have not been fully clarified. Many evidences support the idea that production of Reactive Oxygen Species (ROS) and oxidative stress cause cell apoptosis and necrosis and cardiac myocyte loss that is a principal mechanism of doxorubicin-induced cardiotoxicity (13).

Oxidative stress occurs when production of ROS surpasses the capacity of antioxidant defense systems (glutathione peroxidase, catalase, SOD) (13). Oxidative stress is related with poor outcomes in cardiovascular diseases (14). Feeble antioxidant ability in cardiomyocytes may be a cause responsible for their great sensitivity to oxidative injury (15) A favorable method to cardioprotection is the employ of pharmacological approaches to decrease oxidative stress in the heart (13). In this study, we used H9c2 cells as a pharmacological model to estimate the possible protective effect of HSE on cardiomyocytes. The results indicated that HSE has protective effect on H9c2 cells against DOX-induced oxidative stress. H9c2 cells are morphologically alike to immature embryonic cardiomyocytes. Because these cells preserve hormonal and electrical signal pathways found in adult cardiac cells (16), they are a suitable model for studying oxidative stress-induced cardiomyocyte injury (17). In this model, DOX significantly decreased the cell viability and increase apoptotic rate. As in this model oxidative stress has very harmful effects on cardiac cells, natural products with antioxidant activity can improve or prevent cell damage. Many antioxidants test successfully in this model. It revealed that thymoquinone has protective effect on doxorubicin-induced cardiotoxicity (18). Also, it was shown that gingerol protected cardiomyocytes against DOX-induced toxicity through its antioxidant properties (19). Furthermore, other researches showed that *Glycyrrhiza* could improve rabbit myocardial ischemia-reperfusion injury through P38MAPK pathway (20). Also it has been shown that extract of *Glycyrrhiza* ameliorate doxorubicin-induced cell apoptosis in H9c2 cells (21). For the first time, the protective effect of *H.sabdariffa *on DOX- induced cell death was studied in H9c2 cells. In this research, pretreatment with HSE made cell protection in a concentration dependent manner. HSE could increase cell viability and decrease cell apoptosis. These effects may be partly attributed to antioxidant activity and great capacity of HSE for scavenging free radicals. 

Recent studies have shown that *H. sabdariffa* has bioactive properties that may have a fundamental function in preventing chronic diseases such as diabetes, atherosclerosis hypertension, cardiovascular disease, and reduction high blood cholesterol (22). Also, *H. sabdariffa *has strong antioxidant activity. It is including polyphenols, anthocyanins and flavonoids which have antioxidant activity (23). The antioxidant mechanism of HSE is due to scavenge reactive oxygen and free radicals, inhibition of xanthine oxidase activity, reduction of lipid peroxidation, and elevation of antioxidant enzymes activity (24). *In-vitro* and *in-vivo* studies have revealed antioxidant activity of *H. sabdariffa*. It decreased oxidative stress in rat primary hepatocytes and scavenged free radicals (9). Oboh reported that *H. sabdariffa* has protective effect on Pro-oxidant-induced lipid peroxidation in isolated Brain of rat (10). The mentioned useful effects of *H. sabdariffa* were observed for both ethanolic and water extracts from flowers, leaves, or seeds (24). So, the protective effects of *H. sabdariffa *on DOX are through reduction of oxidative damage. In this study, HSE improved cell viability and decreased cell apoptosis against DOX toxicity in H9c2 cells via its antioxidant properties. Recent studies have revealed that HSE makes apoptosis by p38 MAPK and JNK stimulation, and translocation of cytochrome c from the mitochondria to the cytosol and caspase cascade activation (25). Polyphenol-rich HSE induce cell apoptosis in gastric carcinoma cells by activation of p38/Jun/FasL signaling and steadying of p53, causing a rise in Bax and cytochrome c release, and leading to the activation of caspase-3 (26). *H. sabdariffa* anthocyanin-rich extract induces cell apoptosis in promyelocytic leukemia cells by amplified phosphorylation of p38 and c-Jun, cytochrome c release, and expression of tBid, Fas, and FasL (27). So, observed apoptosis in H9c2 cells could share to the mentioned mechanisms which require more studies.

## Conclusion

We conclude that *H. sabdariffa *has protective effects on DOX-induced cytotoxicity in H9c2 cells through its antioxidant activity and antiapoptotic properties. But more investigations are needed to elucidate the probable underlying mechanisms of these valuable effects.
